# Increasing antigen presentation on HSV-1-infected cells increases lesion size but does not alter neural infection or latency

**DOI:** 10.1099/jgv.0.001059

**Published:** 2018-04-05

**Authors:** Tiffany A. Russell, Thilaga Velusamy, Yeu-Yang Tseng, David C. Tscharke

**Affiliations:** John Curtin School of Medical Research, The Australian National University, Canberra, ACT, Australia; ^†^​ Present address: Department of Microbial Sciences, University of Surrey, Guildford, UK.

**Keywords:** herpes simplex virus, HSV, CD8+ T cells, antigen presentation, immunopathology, latency

## Abstract

CD8^+^ T cells have a role in the control of acute herpes simplex virus (HSV) infection and may also be important in the maintenance of latency. In this study we have explored the consequences of boosting the efficacy of CD8^+^ T cells against HSV by increasing the amount of an MHC I-presented epitope on the surface of infected cells. To do this we used HSVs engineered to express an extra copy of the immunodominant CD8^+^ T cell epitope in C57Bl/6 mice, namely gB_498_ (SSIEFARL). Despite greater presentation of gB_498_ on infected cells, CD8^+^ T cell responses to these viruses in mice were similar to those elicited by a control virus. Further, the expression of extra gB_498_ did not significantly alter the extent or stability of latency in our mouse model, and virus loads in skin and sensory ganglia of infected mice were not affected. Surprisingly, mice infected with these viruses developed significantly larger skin lesions than those infected with control viruses and notably, this phenotype was dependent on MHC haplotype. Therefore increasing the visibility of HSV-infected cells to CD8^+^ T cell attack did not impact neural infection or latency, but rather enhanced pathology in the skin.

## Introduction

Herpes simplex virus (HSV) is a successful pathogen with worldwide distribution that causes a lifelong, though frequently asymptomatic, infection. HSV infection is characterised by a lytic phase that precedes the establishment of latency within the host. During latency, HSV DNA persists in neurons in the absence of a detectable infectious virus, but the virus can periodically reactivate from this state to cause renewed lytic infection that may lead to virus shedding and disease. Adaptive immune responses, including CD8^+^ T cells, are required for the control of acute HSV infection allowing the host to survive and harbour latent infection [1–5].

The role of CD8^+^ T cells in HSV infection is further highlighted by the expression of the immunomodulator ICP47 by HSV. ICP47 reduces the presentation of viral epitopes to CD8^+^ T cells by inhibiting the transporter associated with antigen processing (TAP) [6–8]. ICP47 has poor affinity for mouse TAP and was initially considered to be ineffective in this species, however deletion of ICP47 from HSV reduces the ability of the virus to invade the brain in mice [9–11]. These conflicting reports leave the extent of ICP47 activity in mouse models compared with human infection an open question.

Beyond the acute infection, a role for CD8^+^ T cells in latency has been suggested both in mouse models and in human studies [12–14]. This role has led to proposals that immunotherapy might be an effective treatment for HSV and so the consequences of increasing any parameter associated with CD8^+^ T cell immunity to HSV is of interest [14]. Therefore, we wanted to explore the consequences of making HSV-infected cells more visible to CD8^+^ T cells by increasing the amount of viral antigen presented on MHC class I.

One potential method of increasing the amount of a particular CD8^+^ T cell epitope that is presented on infected cells is to express it as a minimal epitope construct (minigene). An epitope minigene comprises the sequence that encodes a minimal immunogenic peptide, preceded by a start codon. The short polypeptides produced are efficiently presented on MHC I, presumably because they do not require any processing or trimming [15–17]. A variation on this method is to add an endoplasmic reticulum (ER)-targeting sequence to the front of the minimal epitope, such that presentation no longer requires processing by proteases, or transport by TAP [18]. In the case of HSV, such a construct would be expected to circumvent any inhibition by ICP47.

Here we employed these methods to make HSV-1s that present more of the immunodominant gB_498_ peptide on MHC I and tested the impact of this increased expression on the pathogenesis of infection.

## Results

### Generation of recombinant HSV-1s with an additional copy of gB_498_ as a cytosolic or ER-targeted minigene

**Fig. 1.**
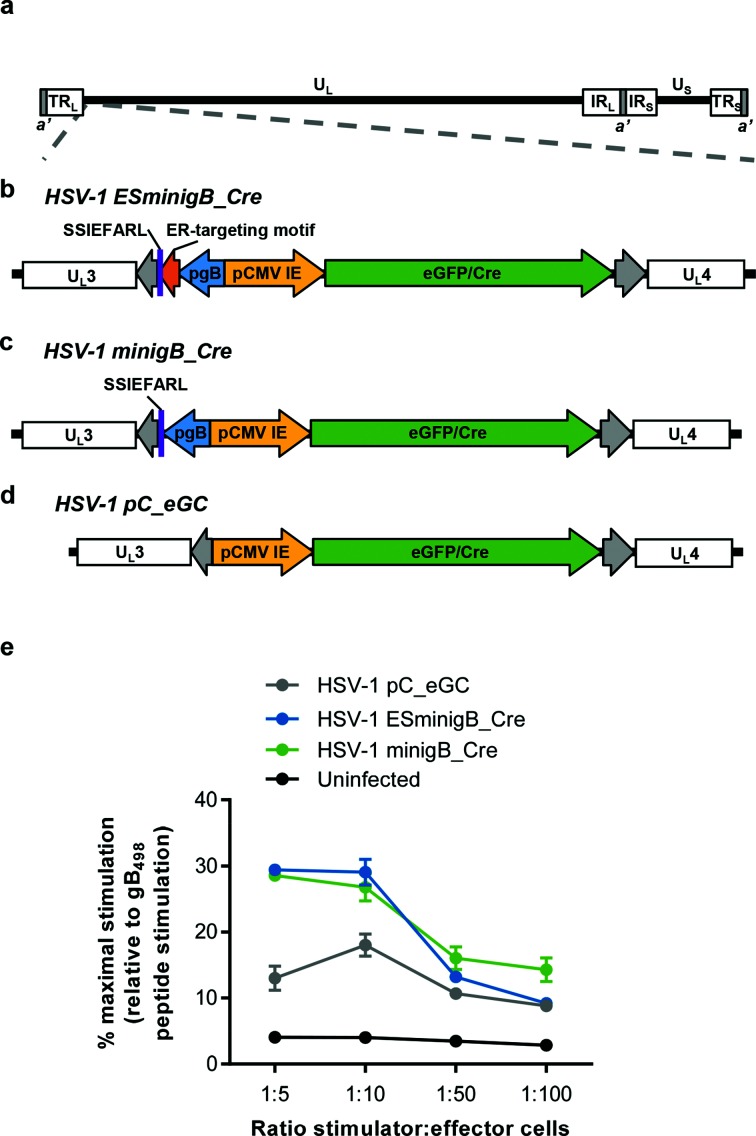
Design of recombinant viruses containing an additional copy of the gB epitope. (a) Schematic representation of the HSV-1 genome with the location of U_L_3 and U_L_4 indicated, to scale. (b) Schematic representation of the CMV IE promoter eGFP/Cre expression cassette inserted into the intergenic space between U_L_3 and U_L_4 in HSV-1 pC_eGC, with an ER-targeting motif (shown in orange) and the minimal gB_498_ minigene (shown in purple) under the control of the gB promoter. (c) As in (b) but with the minimal gB_498_ minigene only (shown in purple) under the control of the gB promoter. (d) Schematic representation of the control virus HSV-1 pC_eGC. (e) 293KbC2 cells were infected with HSV-1 pC_eGC (shown in grey), HSV-1 ESminigB_Cre (shown in blue) or HSV-1 minigB_Cre (shown in green) for 6 h, or were mock infected with PBS (shown in black) and were then cocultured with HSV-2.3.2E2 cells for 12 h at the indicated stimulator: effector ratio. To serve as a positive control, cells were stimulated with 0.125 µM gB_498_ peptide and cocultured with HSV-2.3.2E2 cells. Cells were lysed and assayed for β-gal expression using ONPG, and percent stimulation calculated relative to maximal gB_498_ peptide stimulation. Each stimulation was performed in triplicate and results presentation are mean±sem. The results shown are representative of three independent experiments.

Initially, a recombinant HSV-1 was constructed with an additional copy of the gB_498_ epitope as a minigene with an ER-targeting motif, and named HSV-1 ESminigB_Cre, Fig. 1(b). An *eGFP/cre* fusion gene under the control of the cytomegalovirus immediate early (CMV IE) promoter was also introduced into this virus simultaneously. HSV-1 ESminigB_Cre was then modified to remove the ER-targeting motif, so this second new virus contains a cytosolic gB_498_ epitope (named HSV-1 minigB_Cre), Fig. 1(c). These viruses were compared to HSV-1 pC_eGC, Fig. 1(d), which contains the *eGFP/cre* fusion gene under the control of the CMV IE promoter, but contains only the native gB_498_ epitope located within the gB protein.

### Adding a gB_498_ minigene to HSV-1 enhances presentation on infected cells

The short peptides expressed as minigenes are too small and short in half life to allow direct detection. Therefore to confirm that the gB_498_ minigenes are expressed and able to enhance presentation on MHC-I, an indirect *in vitro* antigen presentation assay was established. This assay uses a T cell hybridoma (HSV-2.3.2E2) that recognises the gB_498_ epitope presented on the MHC I allomorph H-2K^b^ and responds by expressing *LacZ* (under control of an IL-2 promoter) [19]. The hybridoma was co-cultured with HSV-1-infected 293KbC2 cells and activation, quantified by β-galactosidase (β-gal) activity, was used to infer the amount of gB_498_ epitope being presented. In these assays, HSV-1 ESminigB_Cre- and HSV-1 minigB_Cre-infected cells stimulated more β-gal activity from the gB_498_ hybridoma than the control HSV-1 pC_eGC, Fig. 1(e). However, there was no significant difference observed between the two viruses that expressed additional copies of the gB_498_ epitope. Therefore we concluded that regardless of whether gB_498_ was targeted to the ER or not, an additional copy resulted in enhanced presentation of this epitope on infected cells.

### The magnitude of CD8^+^ T cell responses are not changed by addition of a gB_498_ minigene to HSV-1

**Fig. 2.**
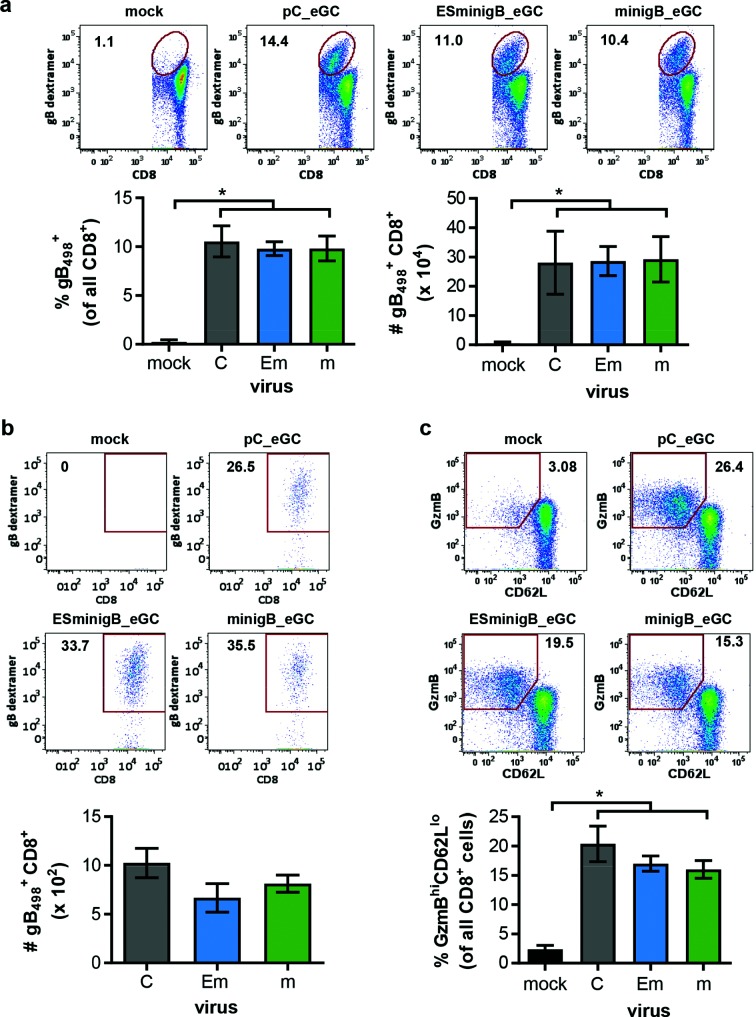
Addition of gB_498_ minigenes to HSV-1 does not affect the magnitude of CD8^+^T cell responses. Groups of C57Bl/6 mice were tattoo infected with HSV-1 as indicated (HSV-1 pC_eG, C, control; HSV-1 ESminigB_Cre, Em; HSV-1 minigB_Cre, m), or PBS (mock), and at 7 days p.i. the magnitude of the CD8^+^ T cell response in spleen (a, c) and DRG (b) was measured by gB_498_ dextramer (a, b) or CD62L and GzmB staining (c). Representative flow cytometry plots are shown at the top of each panel. Underneath data for 5–6 mice from two experiments are shown: (a) gB_498_-specific CD8^+^ T cells in the spleen as percent (left) and total number (right); (b) total numbers of gB_498_-specific CD8^+^ T cells in the DRG; (c) activated (GzmB^hi^CD62L^lo^) CD8^+^ T cells in the spleen as a percent. Mean±sem are shown. Means were compared by one way ANOVA and no significatant differences were found.

Next we wanted to examine whether enhanced presentation on HSV-infected cells might lead to a change in the magnitude of gB_498_-specific CD8^+^ T cell responses in mice. To do this, mice were infected with the viruses by tattoo on the flank and gB_498-_specific CD8^+^ T cell responses measured in the spleen and infected dorsal root gangia (DRG) seven days later, Fig. 2(a, b). There were no significant differences in the size of the gB_498_-specific CD8^+^ T cell responses at either site across the three viruses. We also examined the fraction of splenic gB_498_-specific CD8^+^ T cells that were positive for granzyme B to infer their functional capacity and found no differences across viruses (not shown). Finally, we used staining for granzyme B and CD62L as a measure of the total CD8^+^ T cell response (irrespective of specificity) in the spleen [20] and also found no difference between the control and viruses expressing additional gB_498_, Fig. 2(c).

### The extent and stability of latency is unaltered in mice infected with HSV-1 expressing a gB_498_ minigene

**Fig. 3.**
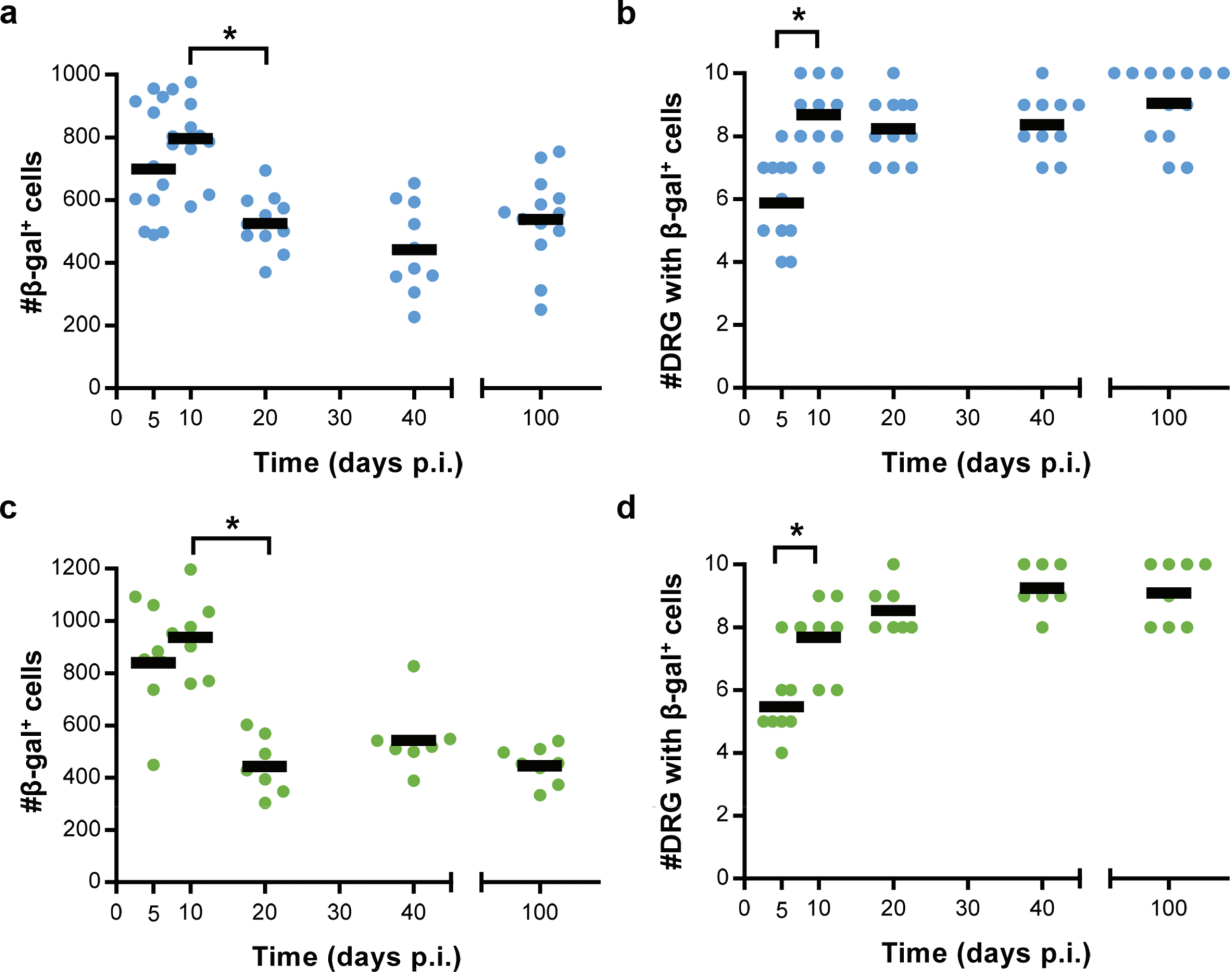
HSV-1 ESminigB_Cre and HSV-1 minigB_Cre establish stable latency. Groups of 3–5 ROSA26 mice were tattoo infected with HSV-1 ESminigB_Cre (a, b) or HSV-1 minigB_Cre (c, d) as indicated, and at the days indicated, mice were culled and the innervating DRG were removed and processed for determination of β-gal expression. Both (a, c) the total number of β-gal^+^ cells per mouse and (b, d) the number of DRG per mouse containing at least one β-gal^+^ cell are shown, with each point indicating a single mouse, and the black bar indicating the mean value. Results are pooled from two independent experiments (*n*=8 per time point). Statistical significance was determined by a one-way ANOVA with Bonferroni’s post-test to make pairwise comparisons, with key statistical differences indicated (*P<0.05).

CD8^+^ T cell responses have been shown to be important in control of acute HSV infection in the DRG and have been implicated in the control of latency [21–27]. For this reason we were interested to determine whether latency establishment or stability might be altered by increasing expression of gB_498_. All the viruses used here express Cre recombinase under the cytomegalovirus immediate early (CMV-IE) promoter enabling us to use the ROSA26 Cre reporter mouse system [26, 28]. In this model, Cre under the CMV-IE is expressed in all virus-infected cells, regardless of whether the outcome is lytic or latent infection, leading to β-gal expression from the ROSA26 genome. The result is an indelible marking of all surviving neurons with a history of HSV infection that can then be counted by appropriate staining and microscopy. In ROSA26 mice infected with HSV-1 ESminigB_Cre, or minigB_Cre, Fig. 3(a, c), latency is established in a similar number of cells as that previously reported using the control virus HSV-1 pC_eGC [26, 27]. In addition, the spread of viruses also appears similar, as determined by the number of DRG that contain at least one β-gal^+^ cell, Fig. 3(b, d). Finally, the total number of β-gal^+^ cells is similar across days 20, 40 and 100 p.i., in mice infected with these viruses, indicating that HSVs expressing additional gB_498_ maintain stable latency. The one parameter we noted was different in these experiments, compared with our previous published work using HSV-1 pC_eGC, was that the number of β-gal^+^ cells did not rise significantly between days 5 and 10. For this reason we examined the acute infection with these viruses more closely.

### The impact of expressing a gB_498_ minigene on the acute HSV-1 infection *in vivo*

**Fig. 4.**
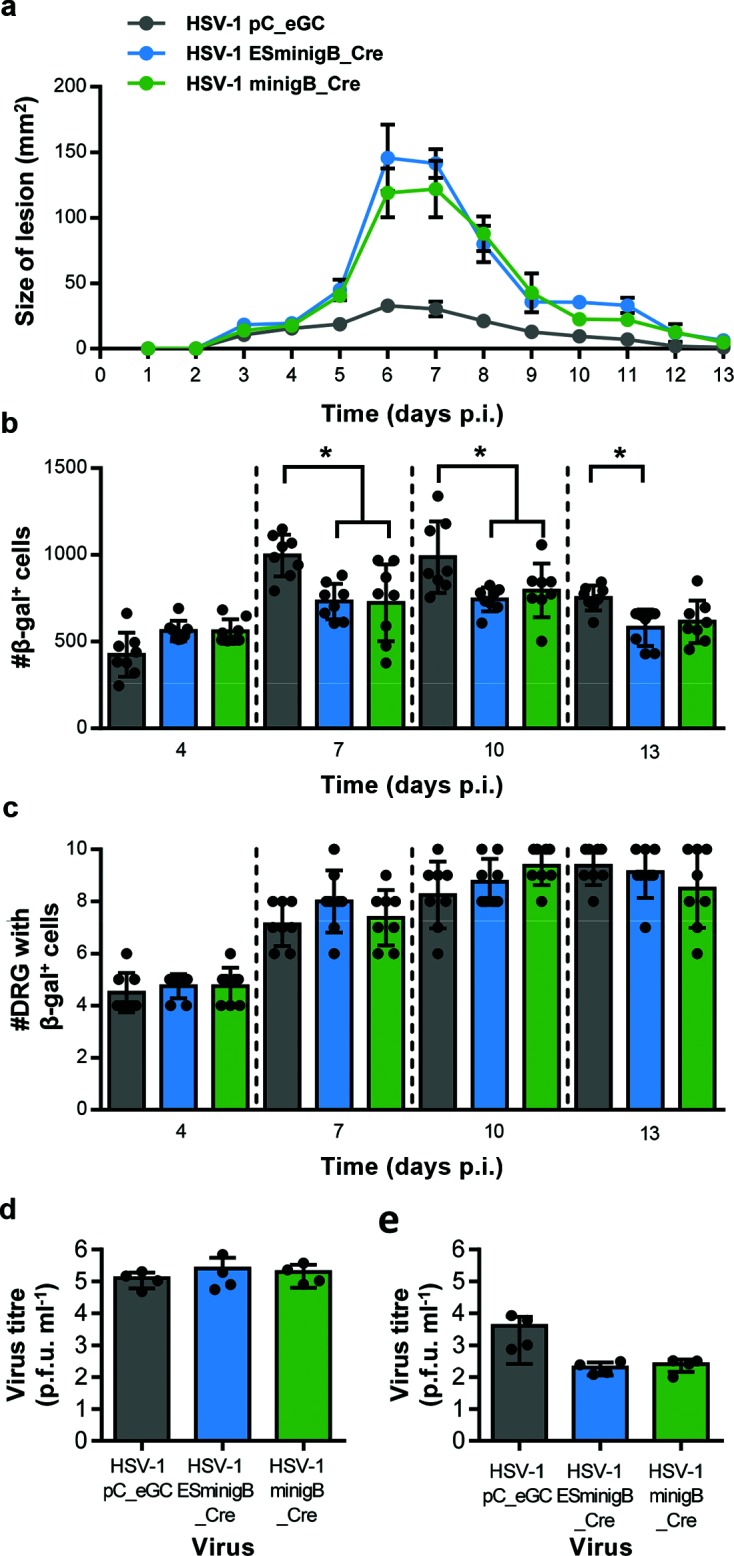
Lesion size, virus growth and neural infection in mice infected by HSV-1 with gB_498_ minigenes. Groups of ROSA26 mice were tattoo infected with the indicated viruses, and lesion size measured daily (a). At the days indicated, mice were culled and the innervating DRG were removed and processed for determination of β-gal expression. Both (b) the total number of β-gal^+^ cells per mouse and (c) the number of DRG per mouse containing at least one β-gal^+^ cell are shown, with each point indicating a single mouse, and the column indicating the mean value. Groups of four C57Bl/6 mice were infected with the indicated virus and at 5 days p.i. were culled and the virus titres in the skin (d) and DRG (e) were determined. Results are pooled from two independent experiments (*n*=8 per time point). Statistical significance was determined by a one-way ANOVA with Bonferroni’s post-test to make pairwise comparisons, with key statistical differences indicated (*P<0.05).

We next decided to investigate the impact of enhanced gB_498_ antigen presentation on acute herpes simplex in mice after flank infection more broadly. To do this we examined (i) skin lesions, (ii) numbers of neurons marked in ROSA26 mice and (iii) amounts of infectious virus in the skin and DRG. (i) The most striking finding was that infection with HSV-1 ESminigB_Cre or HSV-1 minigB_Cre resulted in the formation of significantly larger skin lesions, Fig. 4(a). However, the larger lesions were not associated with an increase in other clinical signs of infection (posture, activity and fur texture; data not shown). (ii) There were significantly fewer β-gal^+^ cells in the mice infected with HSV-1 ESminigB_Cre or HSV-1 minigB_Cre relative to HSV-1 pC_eGC at days 7, 10 and 13 p.i., Fig. 4(b). However, the spread of the virus up and down the spinal column, as determined by the number of DRG that contain at least one β-gal^+^ cell, was similar for all viruses on all days, Fig. 4(c). (iii) Despite the changes noted above, at five days after infection there were no significant differences in virus titre amongst the viruses, either in the skin or in DRG, Fig. 4(d, e). Having stated the lack of statistical significance, we note that the mean titres in DRG were lower for the viruses expressing the gB_498_ minigenes and this might be construed to be consistent with less marking of neurons at days 7 and 10 after infection.

### The impact of expressing a gB_498_ minigene from HSV-1 on skin lesions depends on the MHC anchor residue and strain of mice

**Fig. 5.**
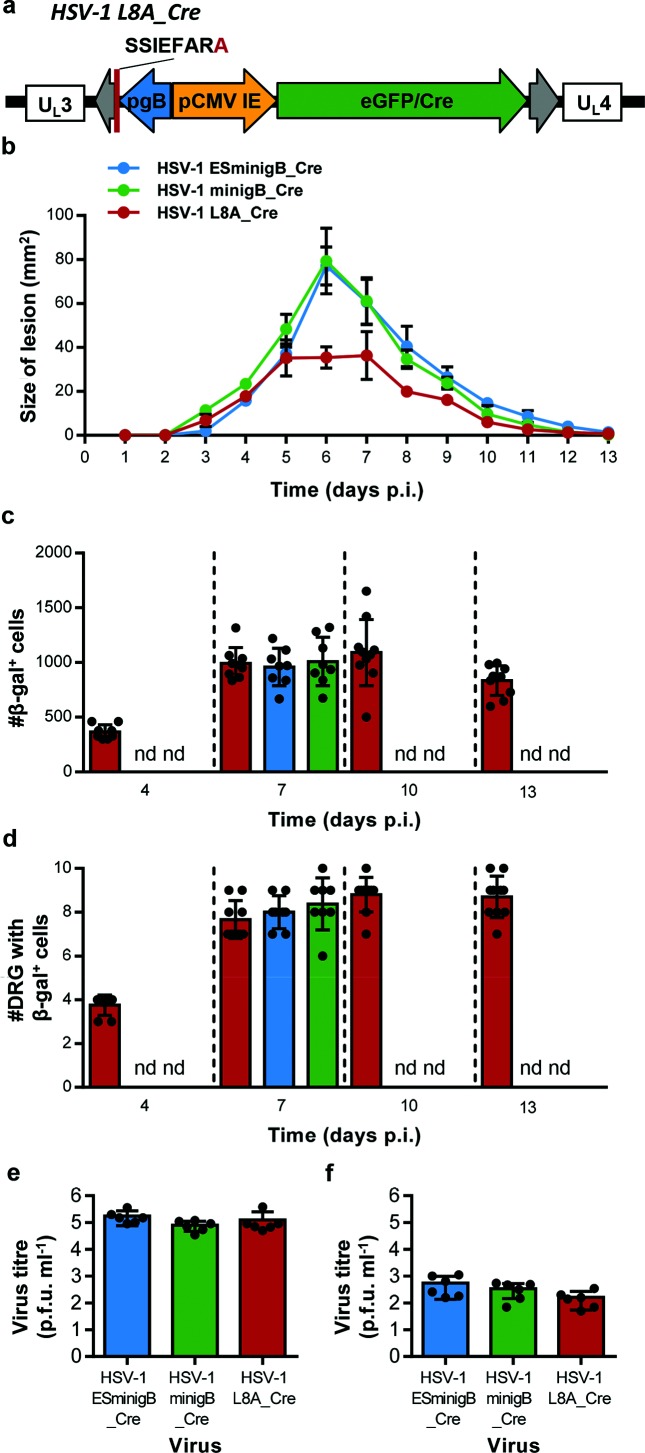
Increased lesion size is the only consistent finding associated with addition of gB_498_ minigenes to HSV-1. (a) Schematic representation of the CMV IE promoter eGFP/Cre expression cassette inserted the intergenic space between U_L_3 and U_L_4 in HSV-1 pC_eGC, with the alternate L8A substituted gB_498_ minigene (shown in red) under the control of the gB promoter. Groups of ROSA26 mice were tattoo infected with the indicated viruses, and lesion size measured daily (b). At the days indicated, mice were culled and the innervating DRG were removed and processed for determination of β-gal expression. Both (c) the total number of β-gal^+^ cells per mouse and (d) the number of DRG per mouse containing at least one β-gal^+^ cell are shown, with each point indicating a single mouse, and the column indicating the mean value (nd=not determined). Groups of four C57Bl/6 mice were infected with the indicated virus and at 5 days p.i. were culled and the virus titres in the skin (e) and DRG (f) were determined. Results are pooled from two independent experiments (*n*=8–10 per time point). Statistical significance was determined by a one-way ANOVA with Bonferroni’s post-test to make pairwise comparisons, with key statistical differences indicated (*P<0.05).

To improve these findings and to confirm they were linked to enhanced presentation of the gB_498_ peptide and not the general construction of the particular recombinant viruses used, we repeated the experiments with a more ideal control virus. This virus, named HSV-1 L8A_Cre, contains the *eGFP/Cre* fusion gene under the control of the CMV IE promoter, along with a modified copy of the gB_498_ minigene, Fig. 5(a). This modified gB_498_ minigene encodes an alanine rather than leucine as the last residue of the peptide, abolishing binding to MHC-I K^b^ and therefore presentation to CD8^+^ T cells [29].

With this new control in hand we repeated the experiments described above. (i) Confirming previous results, lesion size was found to be significantly larger on mice infected with the gB_498_ minigene viruses compared with the L8A control, Fig. 5(b). Other clinical signs were not different amongst the viruses (data not shown). (ii) Marking of neurons in ROSA26 mice by HSV-1 L8A_Cre over the acute infection showed a similar general pattern as published previously and shown in Fig. 4 for the other viruses used here Fig. 5(c, d). However, a direct comparison of this control virus and HSV-1 ESminigB_Cre or HSV-1 minigB_Cre found that equal numbers of neurons were marked on days 7 p.i. This is in contrast to findings with the original control virus. (iii) The virus loads were also similar across these three viruses, both in the skin and in the DRG. Therefore only the skin lesion phenotype remained as a consistent finding associated with expression of gB_498_ minigenes when the L8A virus was used as a control.

**Fig. 6.**
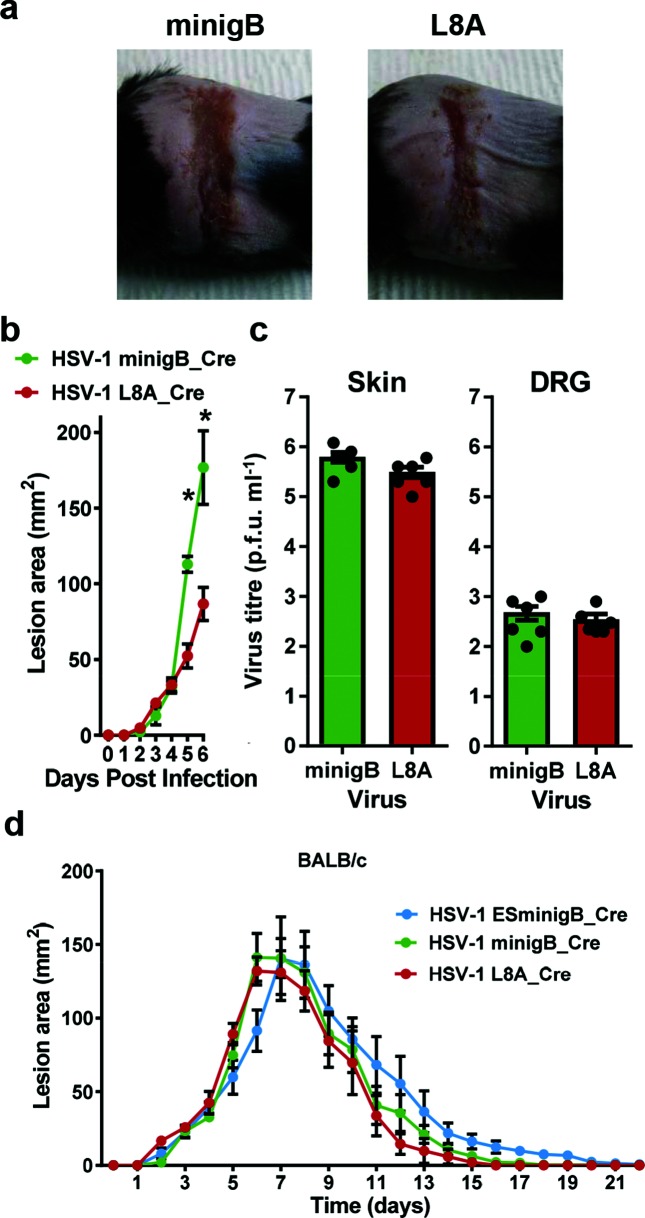
Increased gB_498_ does not affect virus titres in C57Bl/6 mice at day 6, or lesions in BALB/c mice. Groups of six C57Bl/6 mice were tattoo infected with the indicated viruses. Representative photographs of lesions on day 6 are shown (a) with the lesion size estimates for the first six days (b) and significance is shown (*P<0.05). Virus titres in the skin and DRG (c) were determined on day 6. (d) Groups of six BALB/c mice were tattoo infected with the indicated viruses and lesion sizes measured. The lesions were not significantly different across viruses by ANOVA.

In the experiment using L8A as a control, the difference in lesion size was less striking and only significant at day 6. To ensure that this result was robust, we repeated the comparison of HSV-1 minigB_Cre and HSV-1 L8A_Cre observing lesions until day 6 when virus titres were determined in skin and DRG. In this experiment, the difference in lesion size was significant both on day 5 and on day 6. At day 6, when the difference in lesions was largest, Fig. 6(a, b), we also examined virus loads in the skin and found no difference between the viruses, Fig. 6(c). The loss of the skin phenotype with a single amino acid change that is known to disrupt binding of the gB_498_ peptide to MHC class I strongly implicates antigen presentation and CD8^+^ T cells in the mechanism. However, to test this further we examined the lesions caused by our viruses on BALB/c mice that have the H-2^d^ MHC haplotype that does not present the gB_498_ peptide, Fig. 6(d). Unlike the experiments with C57Bl/6 mice, no difference in lesions was found across viruses that express the additional gB_498_ peptide and the L8A control in BALB/c mice.

## Discussion

The overall aim of this study was to examine the consequences of enhancing the visibility HSV-1-infected cells to CD8^+^ T cells *in vivo*. We did this by expressing two minigene versions of gB_498_, one that would be expected to require transport by TAP and another that would be directly inserted into the ER. The decision to include the ER-targeted form was because the extent to which ICP47 might be active in blocking murine TAP remains unknown. On the one hand, biochemical studies suggest that the affinity of ICP47 binding to mouse TAP is very poor, on the other deletion of this gene reduced spread of HSV-1 to the central nervous system [9–11]. Here we found no difference in presentation of gB_498_ *in vitro* using a T cell hybridoma-based assay, irrespective of whether we used an ER targeting motif or not, which was particularly surprising because we used human cells (derived from 293A). We have not tested otherwise for the TAP-independence of the ER targeted peptide, so it remains possible that in HSV-ESminigB_Cre this was not functioning as designed. Alternatively it might be that exceptionally high levels of peptide are generated by the cytoplasmic construct and enough is transported even in the presence of ICP47 to allow high levels of activation of the T cell hybridoma. We have mass spectrometry data to show that antigen presentation is not entirely blocked by ICP47 in the 293KbC2 cells used here and the extent of the block is somewhat epitope dependent (TV, DCT unpublished). It has also been noted by others that upregulation of TAP as a result of interferon-γ exposure can temper the effect of ICP47 [30]. Never-the-less, both minigene viruses presented more gB_498_ than the parent HSV and they behaved identically in mice *in vivo*, acting as independently derived control viruses.

HSV-1 ESminigB_Cre and HSV-1 minigB_Cre activated similar levels of gB_498_-specific CD8^+^ T cells in spleen and DRG, compared with the control virus. This is despite greater presentation of gB_498_ on infected cells. Rather than being contradictory, this was expected because of two observations in the literature: first, CD8^+^ T cell responses to cutaneous HSV-1 infection of mice are thought to be largely cross-primed, which means that non-infected dendritic cells pick up exogenous antigen for processing and presentation on MHC I [19, 31–33]. Second, epitope minigenes are poorly presented by cross presentation [34–36]. In support of this interpretation, rather than there being any unique properties of gB_498_ minigenes, expression of minigene gB_498_ from vaccinia virus, which is expected to directly prime CD8^+^ T cells, leads to higher responses to this peptide than the full gB protein [37].

Studies going back more than two decades have suggested that the main role for CD8^+^ T cells in mouse models of HSV is protection of sensory neurons, so we expected that any impact of increasing presentation of gB_498_ would be seen in DRG [21]. This included potential effects on the stability of latency, because CD8^+^ T cells have been suggested to play a role in maintaining the latent state [24, 38, 39]. Despite the historic view that the nervous system is immunopriviledged, a role for adaptive immunity is reasonable because the presence of activated CD8^+^ T cells and evidence of lytic gene expression suggest that antigen presentation is possible even during latency [25–27, 40, 41]. However, taking all of our results together, we found no difference in any parameter of infection of DRG or latency in mice infected with viruses that had gB_498_ minigenes. These findings echo those of a study that had the potential to improve antigen presentation by expression of an MHC I heavy chain from HSV-1 [42]. There are several caveats that should be acknowledged in our experiment. First, we cannot be entirely sure that the minigenes were expressed in neurons. As noted above, these short polypeptides are too small and evanescent to allow detection. However, using the same promoter driving Cre from the same locus in the HSV-1 genome we have recently shown that many neurons are marked in ROSA26 mice, both during the acute infection and during latency [27]. Second, even if the genes were expressed, they might not lead to more presentation of gB_498_ on neurons, or the amount presented from the endogenous copy of the epitope may already be adequate to ensure a maximum response in DRG. Third, if the action of CD8^+^ T cells is non-lytic suppression of virus or reactivation as has been suggested [21, 24], this would be undetectable in our model. Finally, the model used is one that mimics stable latency so impacts on reactivation were not assessed beyond noting that virus could be produced at typical rates after explant culture of latently-infected DRG (not shown). A more physiologically relevant model of reactivation that occurs in the context of the CD8^+^ T cell response [43, 44] may demonstrate some effect of enhanced gB_498_ presentation. Taking these into consideration our experiment cannot be interpreted as bearing significantly on the question of the role of CD8^+^ T cells in HSV-1 latency and reactivation.

Where we failed to find a phenotype for our viruses in DRG, there was a striking increase in the size of lesions caused by those viruses incorporating a gB_498_ minigene. Our method of measuring size incorporates an estimate of the total area where there are lesions as well as the fraction of skin in this area that is affected. This is important because in some mice infected with control viruses, the lesion does not become confluent across the whole dermatome. In the case of mice infected with the minigene viruses, the lesions covered a larger area as well as being more confluent. Of note, the amounts of infectious virus in the skin of these mice were always the same, even at day 6 when there was a difference in lesion size in all experiments, suggesting the cause of the larger lesions was immunopathology. This is supported by the L8A control, which differed from the cytoplasmic minigene by only a single amino acid and the lack of a difference in lesion size in BALB/c mice. This implicates presentation on MHC class I and therefore the action of CD8^+^ T cells, at least locally in the skin, in the pathology of the larger lesions. The role of proinflammatory processes in modulating the progression and resolution of HSV skin lesions has been previously noted, with a clinical trial showing an increased efficacy of the commonly used HSV topical antiviral acyclovir when combined with hydrocortisone cream [45]. Any mechanism remains highly speculative, but we suggest that increasing the amount of antigen may increase (a) the recruitment/retention of CD8^+^ T cells, (b) their range of targets, perhaps including uninfected cells by ‘cross-dressing’, and/or (c) the amount of proinflammatory mediators they release in infected skin.

In conclusion we find that our strategy for making HSV-infected cells more easily targeted by CD8^+^ T cells has not impacted any parameter of neural infection, but instead was associated with increased pathology in the skin. We suggest that more consideration of potential unexpected immunopathology may be needed when pursuing CD8^+^ T cell-based immunotherapies for HSV-1.

## Methods

### Viruses and cell lines

All viruses were grown and titrated on Vero cells (ATCC CCL-81). The immortalised Vero cell line, and HSV-2.3.2E2 hybridoma [19] used for the antigen presentation assay, were maintained in Minimal Essential Medium (Life Technologies) supplemented with 2 or 10 % heat inactivated fetal calf serum (FCS; Serana), 5 mM HEPES, 4 mM l-glutamine and 50 µM 2-mercaptoethanol (Life Technologies). All transfections were carried out in 293A cells using Lipofectamine 2000 (Life Technologies). Cells of the 293KbC2 line [46] were used for the antigen presentation assay and were maintained in Dulbecco’s Modified Eagles Medium (DMEM; Life Technologies) supplemented with 2 or 10 % heat inactivated FCS (Serana) and 2 mM l-glutamine (Life Technologies).

HSV-1 pC_eGC contains an eGFP/Cre fusion gene under the control of the (CMV IE) promoter located in the intergenic region between U_L_3 and U_L_4 of HSV-1 KOS [47].

### Plasmid construction

All sequence references below are to the HSV-1 KOS genome accession JQ673480. Briefly, the plasmids used to construct the recombinant viruses all contained the eGFP/Cre fusion gene (amplified from pIGCN21 [48]) under the control of the CMV IE promoter (amplified from pTracer CMV/bsd; Life Technologies), with the bovine growth hormone (BGH) polyA termination sequence (amplified from pTracer CMV/bsd; Life Technologies), inserted into the SpeI site of pU3.2kbF [47]. To construct pESminigB_Cre, an ER-targeted gB minigene [49] under the control of the gB promoter sequence (HSV-1 KOS 55985-56282) followed by the SV40 polyA sequence was inserted into the NotI site of pU3.2kbF by In-Fusion cloning (Clontech). pminigB_Cre contains the gB minigene under the control of the gB promoter, but lacking the ER-targeting motif. Finally, pL8A_Cre contains the gB minigene with the L8A substitution under the control of the gB promoter, lacking the ER-targeting motif.

The plasmid pX330 (Addgene plasmid 42230) has been previously described [50]. The plasmid pX330-minigB was constructed by annealing two complimentary oligodeoxynucleotides (CACCGGCCGCGCTGCAGACTGCCGCA and AAACTGCGGCAGTCTGCAGCGCGGCC) and ligating the resulting dsDNA fragment into the BbsI site of pX330.

### Construction of recombinant viruses

To construct HSV-1 ESminigB_Cre, linearised plasmid DNA from pESminigB_Cre was transfected into Vero cells, followed by infection with HSV-1 KOS as previously described [47]. Plaques containing the desired recombinant virus were identified based on eGFP expression and PCR screening, and at least three rounds of plaque purification were carried out.

To construct HSV-1 minigB_Cre and HSV-1 L8A_Cre, transfection of linearised pminigB_Cre or pL8A_Cre with pX330-minigB was performed, followed by infection with HSV-1 ESminigB_Cre as previously described [47]. Desired recombinant virus was identified based on PCR screening, and at least three rounds of plaque purification were carried out. PCR screening and sequencing were used to ensure that the viruses contained the desired modification and were free from parental virus. All viruses grew with normal kinetics after low multiplicity infection of cells in culture and were able to be reactivated from the DRG of latently-infected mice (not shown).

### Antigen presentation assay

Single cell suspensions of 293KbC2 cells were infected with the appropriate virus at a MOI of 5 as previously described [51]. After 6 h, infected cells (stimulators) were mixed with 5×10^4^ HSV-2.3.2E2 hybridoma cells (effectors) at a stimulator to effector ratio of 1 : 5, 1 : 10, 1 : 50 or 1 : 100, in triplicate. Background *LacZ* levels were determined using hybridoma cells alone, while uninfected cells served as stimulators for a negative control, and 293KbC2 cells stimulated with 0.125 µM synthetic gB_498_ (sequence SSIEFARL; GenScript) served as a positive control. After 12 h at 37 °C, 5 % CO_2_, β-gal activity was measured using ortho-Nitrophenyl-β-galactoside (ONPG) as a substrate as previously described [52], reading on a Tecan Infinite M1000 Pro plate reader.

### Mice and infections

Female specific pathogen free C57Bl/6, BALB/c or B6.129S4-Gt(ROSA)26Sor^tm1So^/J (referred to as ROSA26) mice [53] greater than 8 weeks of age were obtained from the Australian Phenomics Facility (Canberra, Australia). Mice were housed and experiments carried out under the oversight of the Animal Ethics and Experimentation Committee of The Australian National University and according to Protocol Numbers A2011.015, A2014.025 or A2017-039. Mice were infected using a flank infection model where 1×10^8^ p.f.u. ml^−1^ virus was introduced into the flank by tattoo as previously described, mice were anaethetised with 1,1,1 tribromoethanol for the infection process [47]. The same virus dose and route of infection were used for all experiments.

### CD8^+^ T cell assays

Mice were culled 7 days p.i. and single cell suspensions of spleens were prepared. To prepare single cell suspensions of DRG, DRG were collected from mice from spinal levels T5 to L1, trimmed of excess tissue, and placed directly into 1 ml of 1 mg ml^−1^ Type IV Collagenase (at least 160 units ml^−1^; Worthington) and 0.03 mg ml^−1^ DNase (at least 600 units ml^−1^; Roche) in DMEM supplemented with 2 % FCS. The DRG were incubated at 37 °C, 200 r.p.m. for 60 min, and gently ground through a 70 µM cell strainer and washed with excess cold PBS containing 2 % FCS.

Cells were stained with one or more of the following mAbs (Biolegend): anti-CD8α-APC-Cy7 (clone 53–6.7), anti-CD62L-FITC (clone MEL-14), anti-CD45.2-BV481 (clone 104), anti-CD4-PE-Cy7 (clone GK1.5) and anti-GzmB-AF647 (clone GB11). This was combined with H-2K^b^/gB-PE dextramer staining as previously described [54]. Cells from the spleen or DRG were fixed with 1 % paraformaldehyde, and data was acquired on a LSRII flow cytometer (BD biosciences). Analysis was performed using Flowjo software (Tree Star), with events gated for live lymphocytes on FSC-A×SSC A plots, with appropriate parameters examined after doublet discrimination.

### Titration of virus from skin and DRG

A 1 cm^2^ portion of skin located over the inoculation site and the DRG found on the ipsilateral side corresponding to spinal levels L1 – T5 were collected 5 days post-infection (p.i.). Skin or DRG were homogenised in MEM supplemented with 2 % FCS. Homogenates were subjected to three cycles of freeze/thawing and infectious virus quantified by plaque assay on Vero cells.

### Histochemical detection of β-gal expression and detection of fluorescence in whole DRG

Mice were culled by CO_2_ asphyxiation and the innervating whole DRG were removed as soon as possible and fixed in 2 % paraformaldehyde/0.5 % glutaraldehyde for 1 h on ice, permeabilised and stained with 1 mg ml^−1^ X-gal for the detection of β-gal expression as previously described [27]. The DRG were then visualised and photographed using an Olympus CKX41 light microscope and Olympus DP20 camera. The number of β-gal^+^ cells was determined with the aid of ImageJ software [55].

### Statistical analysis

Statistical comparisons were performed with the aid of Prism software (version 7.01; GraphPad).
